# Cooling-mediated protection from chemotherapy drug-induced cytotoxicity in human keratinocytes by inhibition of cellular drug uptake

**DOI:** 10.1371/journal.pone.0240454

**Published:** 2020-10-15

**Authors:** Christopher Dunnill, Khalidah Ibraheem, Michael Peake, Myria Ioannou, Megan Palmer, Adrian Smith, Andrew Collett, Nikolaos T. Georgopoulos

**Affiliations:** 1 Department of Biological Sciences, School of Applied Sciences, University of Huddersfield, Huddersfield, United Kingdom; 2 Department of General Surgery, Calderdale and Huddersfield NHS Foundation Trust, Huddersfield, United Kingdom; 3 Institute of Skin Integrity and Infection Prevention, University of Huddersfield, Huddersfield, United Kingdom; Columbia University, UNITED STATES

## Abstract

Chemotherapy-induced alopecia (CIA) represents the most distressing side-effect for cancer patients. Scalp cooling is currently the only treatment to combat CIA, yet little is known about its cytoprotective effects in human hair follicles (HF). We have previously established in vitro human keratinocyte models to study the effects of taxanes and anthracyclines routinely-used clinically and reported that cooling markedly-reduced or even completely-prevented cytotoxicity in a temperature dependent manner. Using these models (including HF-derived primary keratinocytes), we now demonstrate that cooling markedly attenuates cellular uptake of the anthracyclines doxorubicin and epirubicin to reduce or prevent drug-mediated human keratinocyte cytotoxicity. We show marked reduction in drug uptake and nuclear localization qualitatively by fluorescence microscopy. We have also devised a flow cytometry-based methodology that permitted semi-quantitative analysis of differences in drug uptake, which demonstrated that cooling can reduce drug uptake by up to ~8-fold in comparison to normal/physiological temperature, an effect that was temperature-dependent. Our results provide evidence that attenuation of cellular drug uptake represents at least one of the mechanisms underpinning the ability of cooling to rescue human keratinocytes from chemotherapy drug-cytotoxicity, thus supporting the clinical efficacy of scalp cooling.

## Introduction

Although not a life-threatening condition, chemotherapy-induced alopecia (CIA) represents the most distressing side-effect for cancer patients [1] and the fear of hair loss causes severe anxiety in patients [2]. Thus it is important to understand the mechanisms of CIA in order to design safe and effective prevention strategies [3]. Chemotherapy drugs, such as taxanes and particularly anthracyclines, cause CIA as they damage human hair follicles (HF) by mediating direct cytotoxicity in the hair matrix keratinocytes, which are the most rapidly-dividing cell population in the HF [3]. The extent to which various chemotherapy drugs cause CIA differs [4], yet anthracyclines appear to be the biggest inducer of CIA [5].

Scalp cooling is currently the only safe and widely-available preventative treatment to combat CIA in cancer patients [4]. There is increasing clinical evidence that scalp cooling can be an effective way to minimize or even eliminate CIA [6, 7]. Overall the probability that scalp cooling will prevent/reduce CIA is ~50% and this can rise to ~90% depending on the chemotherapy modality used and/or scalp cooling techniques employed [8], whilst the recently-published first multi-centre randomised clinical trial ‘SCALP’ has provided further clinical evidence that supports the ability of scalp cooling to reduce the risk of CIA to ~50% [9]. Therefore, cooling of the scalp is a safe and very promising approach to minimize CIA, and biological research relating to the mechanisms by which cooling prevents chemotherapy drug-mediated cytotoxicity may not only provide scientific support for its ability to cytoprotect, but also assist in the design of strategies to enhance the ability of cooling to prevent CIA.

A number of experimental models are available for studying chemotherapy drug-induced cytotoxicity and CIA, such as in vivo rodent-based models [3], although these have inherent practical and physiological limitations. Ex vivo models based on isolation and culture of human HFs established by Paus and colleagues represent an elegant model that has been used to study CIA caused by a variety of drug types [10–12]. Yet, despite their clear physiological relevance, these models are experimentally laborious, with relatively limited ability for assessment of several parameters simultaneously, which does not permit more systematic and high-throughput investigations.

We previously established a series of in vitro models for the culture of primary and immortalized human keratinocytes under conditions where they adopted a basal highly-proliferative phenotype resembling the rapidly-dividing hair matrix keratinocytes, as well as human hair follicle-derived keratinocytes. Specifically, we used a) normal human epidermal keratinocytes (NHEK), b) human HF-derived keratinocytes, and c) our derivative of the well-characterised keratinocyte line HaCaT which was adapted to serum-free/low-calcium culture conditions (‘HaCaTa’ cells). We employed these models to study the effects of cooling on chemotherapy drug-mediated cytotoxicity. Using a panel of drugs (including both taxanes and anthracyclines) routinely used in the clinic for treatment of cancers such as breast cancer, we demonstrated for the first time that although the drugs were highly cytotoxic, cooling during drug treatment markedly reduced or even completely prevented drug cytotoxicity, in agreement with observations of the ability of scalp cooling to prevent HF toxicity in the clinic [13]. Equally importantly, we provided evidence that the precise cooling conditions (temperature) was a critical factor in determining the overall ability of cooling in rescuing cells from drug-mediated toxicity. The concordance of our findings with clinical observations demonstrated that, despite their reductive nature, these in vitro models were robust and provided experimental support for the clinically-reported cytoprotective role of cooling [13], thus paving the way for further studies on the molecular mechanisms underpinning the cytoprotective effects of cooling.

A number of mechanisms have been proposed to explain the cytoprotective effects of scalp cooling [4], including: i) cooling causes vasoconstriction which can reduce cutaneous perfusion to 20%-40% of normal levels [14], resulting in reduced chemotherapy drug perfusion through the HF; ii) the rate of drug uptake across the plasma membrane is reduced at low temperatures via reduction of active transport or reduced diffusion due to lower cellular activity or kinetic energy and membrane fluidity, respectively; iii) cooling may slow-down the rate of cell division (as cell division is an energy-dependent process); and iv) decreases in the metabolic activity of HF cells by cooling reduces the cytotoxicity of chemotherapy drugs as a range of cellular processes (such as oxidation) decelerate. Most likely, a combination of these mechanisms will render scalp cooling cytoprotective, thus explaining its reported clinical efficacy. Notably, our previous work demonstrated that cooling completely inhibited the cytotoxicity of the anthracycline doxorubicin at concentrations identical to the maximal plasma drug concentration measured during routine infusion with the drug in patients undergoing chemotherapy [13]. Those findings strongly implied that cooling may protect cells by a direct mechanism and not merely by vasoconstriction.

Using our previously-established in vitro models, in this study we aimed to for the first time explore the hypothesis that cooling reduces the entry of chemotherapy drugs into human keratinocytes. We studied doxorubicin and epirubicin as anthracyclines are the strongest inducers of CIA, whilst exploiting the inherent property of doxorubicin [15] and epirubicin [16] to fluoresce in human cells. We found that doxorubicin and epirubicin are detectable in the nuclei of NHEK, HaCaTa and human hair follicle-derived outer root sheath keratinocytes (ORSK cells) and are highly cytotoxic at normal temperature; however, cooling markedly reduced chemotherapy drug cellular uptake, which coincided with its ability to reduce or prevent drug-mediated keratinocyte cytotoxicity. In addition to qualitative detection by fluorescence microscopy, we established a semi-quantitative method to determine cellular drug uptake by using flow cytometry, an approach that allowed us to determine that cooling can reduce drug uptake by up to ~8-fold. Our results suggest that attenuation of drug uptake represents at least one important mechanism by which cooling can protect cells in the HFs from chemotherapy drug-mediated cytotoxicity.

## Materials and methods

### Culture of immortalized keratinocyte cell lines

The HaCaT cell line was originally purchased from the Cell Line Service (CLS, Eppelheim, Germany) as described elsewhere [13] and cultured in DMEM medium supplemented with 10% (v/v) fetal bovine serum (FBS) and 2 mM L-glutamine–all obtained from Sigma (Sigma-Aldrich, Dorset, UK). The HaCaT-derivative keratinocyte line ‘HaCaTa’ was established as detailed previously [13] and cultured in keratinocyte serum free medium (KSFM) supplemented with epidermal growth factor and bovine pituitary extract (referred to as KSFM complete, KSFMc)–all obtained from Thermo Fisher Scientific (Loughborough, UK). All cells were routinely cultured at 37°C in a humidified atmosphere of 5% CO_2_, whereas for cooling experiments the desired temperature was achieved using an LMS Series 1A Cooled Incubator with appropriate CO_2_ provision for the duration of the incubation period (see below). Cells were passaged at ~80–90% confluence by removing media, rinsing with 0.1% EDTA in PBS (w/v) to aid disaggregation, and lifted using trypsin–EDTA solution (Sigma). For HaCaTa cells, the trypsin was inactivated by brief treatment with 5% FBS-containing KSFM medium, before routine culture in KSFMc. HaCaT cells were cultured in standard plasticware, whilst HaCaTa were cultured in Cell Plus (Cell+) plasticware (Sarstedt, Leicester, UK). HaCaT and HaCaTa cell lines were tested for Mycoplasma using the MycoProbe™ Mycoplasma detection assay (R&D Systems).

### Establishment and culture of normal human epidermal keratinocytes

Normal human epidermal keratinocytes (NHEK) were established using skin collected from routine surgical procedures with National Health Service (NHS) Research Ethics Committee (REC) approval and the required, informed written consent from patients with no history of skin malignancy. Full-thickness abdominal skin (~3cm^2^) was collected and initially stored at 4°C in 20ml HBSS medium supplemented with penicillin (100 units/mL)/streptomycin (100 μg/mL) (Thermo Fisher) and Amphotericin B (25 ng/mL) (Sigma). Following removal of adipose and connective tissue by trimming using surgical scissors, the remaining dermis and epidermis were rinsed three times in PBS and then cut to ~4×2 mm/8mm^2^ pieces using a scalpel. These pieces were placed epidermal-side down into a 6-cm petri dish containing 5ml 2.5 mg/ml dispase solution (Sigma) in HBSS supplemented with antibiotics (as above), which was sealed in an airtight container and incubated at 4°C for 12–16 h. Epidermal sheets were then gently removed from the dermis using forceps in ice-cold HBSS; these were then placed into 5 ml 0.25% trypsin/EDTA diluted in HBSS and ‘minced’ for 10-minutes with a scalpel in a 6 cm petri-dish lid at room temperature to create a cell suspension. This was filtered through a 70μm strainer (Corning, supplied by Sigma) using a serological pipette and suspended in 20 ml DMEM/10% FBS to inactivate the trypsin. This was then placed in a 50 ml centrifuge tube and centrifuged at 130 g for 10-minutes. The cell pellet was resuspended in 5 ml EpiLife medium supplemented with HKGS (Thermo Fisher). 10^6^ cells were suspended in a total of 4 ml EpiLife/HKGS containing antibiotics and were seeded in Cell+ T25 flasks (Sarstedt) pre-coated with 200 mg/ml human collagen IV (Merck, Watford, UK) to enhance initial attachment. At this point, cells were classified as passage 0 (p0) and were-medium changed the next day. Upon reaching 60–80% confluency, cultures were passaged (1:3 ratio) in Cell+ T25 flasks (non-collagen-coated). For all experiments, NHEK cells were used at <p5 to avoid the onset of senescence and ensure maximal proliferative capacity.

### Culture of human dermal fibroblasts (HDF)

HDF cells were a gift from Tamás Bíro (University of Debrecen, Hungary) and were cultured in DMEM/10% FBS and 2mM L-glutamine supplemented with penicillin/streptomycin (100IU/100μg/ml) at 3x10^3^ cells/cm^2^ until confluent. For use as feeder cells, confluent HDF cultures were treated with 4 μg/ml Mitomycin C (Acros Organics, supplied by Thermo Fisher) for 24-hours and sub-cultured into 35-mm petri dishes at 3.5×10^4^ cells/dish in a final volume of 2 ml. Feeder layers were used within 2–6 days post-seeding [17].

### Establishment of outer root sheath keratinocyte (ORSK) cultures

For the establishment of ORSK cultures, hair plucking was performed with informed written consent and ethical approval by the School of Applied Sciences Research Integrity and Ethics Committee (SRIEC) and in line with the University of Huddersfield Research Ethics and Integrity Policy and Code of Practice for Research. ORSK cultures were established based on previously detailed methodologies [17–19]. Briefly, 5–10 hair follicles were plucked from consenting donors and, if containing a clearly visible ORS, incubated for 30-minutes at 37°C in 2 ml ORSK medium comprising DMEM supplemented with 10% FBS, 2mM L-glutamine, 100 UI/mL penicillin G, 1 mM ascorbyl-2-phosphate, 2.4 μg/ml adenine, 10 ng/ml epidermal growth factor, 0.4 μg/ml hydrocortisone, 2 nM triiodothyronine, 0.1 nM cholera toxin, 25 μg/mL gentamycin. Follicles were rinsed twice with 1 ml PBS containing 100 μg/ml gentamycin. ORS-containing HFs were incubated with 0.5 ml 0.25% Trypsin-EDTA for up to 40-minutes with vigorous pipetting and vortexing performed at 10-minute intervals until the ORS was disaggregated to provide a suspension of ORSK cells. Trypsin was inactivated by mixing with equal volume of ORSK medium followed by centrifugation and supernatant removal. Cells were then seeded onto HDF feeder cultures in 35-mm petri dishes and incubated for 5–6 days. The 2 ml of ORSK medium was replenished every 2–3 days until subculture was required; for this, ORSK cells were rinsed twice with PBS-gentamycin, and incubated with TrypLE Express (Thermo Fisher) for 5–10 minutes. TrypLE enzyme was then inactivated by dilution in equal volume of PBS and by centrifuging the suspension. Cells were seeded in EpiLife/HKGS medium at 1x10^4^ cells/cm^2^ [20].

### Detection and quantification of cell viability

Keratinocyte cell growth and the effect of chemotherapy drugs on cell viability, was determined using the CellTiter 96® AQueous One cell proliferation assay (Promega, Southampton, UK). For chemotherapy drug experiments, cells were treated with doxorubicin (Doxorubicin hydrochloride, # sc-200923, Santa Cruz Biotechnology) or epirubicin (Epirubicin hydrochloride, # E0550000-1EA, Sigma). HaCaT, HaCaTa and NHEK cells were seeded at 5x10^3^ cells/well and ORSK cells at 8x10^3^ cells/well, respectively, in 100μl culture medium in 96-well plates of either standard or Cell+ plasticware (Sarstedt), as appropriate. 24-hours later, 100μl of doxorubicin or epirubicin solution appropriately diluted in the respective culture medium were added to each well, in order to achieve the desired final drug concentration. Following a 2-hour incubation (drug treatment) at normal (37°C) or at cooling conditions (22 or 18°C), the drug-containing medium was removed and wells were rinsed once with PBS solution at room temperature, before 100μl of fresh medium was added in each well and cells were incubated at 37°C for 72-hours. 20 μl CellTiter reagent was then added to each well and plates were incubated at 37°C for 4-hours. Absorbance was then measured using a FLUOstar OPTIMA (BMG Labtech, Bucks, UK) plate reader at 492 nm wavelength. Percentage (%) cell viability was calculated as previously [13] using the equation: (Abs T/Abs C) x 100, where ‘Abs T’ corresponds to absorbance value for drug-treated cells and ‘Abs C’ is the absorbance value for control cultures.

### Fluorescence microscopy

For doxorubicin uptake visualisation, NHEK and HaCaTa were seeded at a density of 5x10^4^ cells/well on Teflon-coated 12-well glass slides as previously described [21]. After 24-hours, cells were treated for 2-hours with the indicated concentration of doxorubicin in PBS solution at 37°C or at cooling conditions (18°C). Cells were then rinsed twice with PBS, before being fixed using 4% paraformaldehyde in PBS for 15-minutes at room temperature and cell nuclei counterstained for 5-minutes using 0.1 mg/mL Hoechst 33258 (Sigma) as detailed elsewhere [22], prior to mounting and epifluorescence imaging (green channel for doxorubicin and blue channel for Hoechst). For epirubicin uptake visualisation, NHEK and HaCaTa were seeded at a density of 3x10^4^ cells/well in 24-well multi-well plates and 24-hours later cells were treated for 2-hours with the indicated concentration of epirubicin in PBS at 37°C or at cooling conditions (18°C) prior to live fluorescence imaging (green channel). Drug uptake was visualised for doxorubicin by epifluorescence imaging on a Zeiss Axio Imager Z1 and images captured with a Zeiss AxioCam MRm Rev.3 digital camera and analyzed using ZEN software (Carl Zeiss Ltd, Herts, UK), and for epirubicin by live imaging using an EVOS® FLoid® cell‐imaging station (Thermo Fisher) and images analyzed using Image J software.

### Flow cytometry

HaCaT, HaCaTa and NHEK cells were seeded at 3x10^4^ cells/well and ORSK cells seeded at 4.8x10^4^ cells/well (2.5x10^4^/cm^2^) in 500μl culture medium in 24-well plates of standard and Cell+ type plasticware type (Sarstedt), respectively. 24-hours later, 500μl of doxorubicin or epirubicin solution appropriately diluted in the respective culture medium were added to each well, in order to achieve the desired final drug concentration. Cultures treated with the various drug concentrations and incubated at 37°C or at cooling conditions (22 or 18°C), for 2-hours. Following treatment, the drug-containing medium was removed and wells were rinsed once with PBS solution, before harvesting using 200 μl 0.25% trypsin/EDTA diluted in PBS for HaCaT, HaCaTa and NHEK and 1x TrypLE express enzyme for ORSK cells. Cells were counted, washed in FACS buffer as detailed elsewhere [23] and resuspended in 200 μl of ice-cold PBS for flow cytometry analysis. Doxorubicin or epirubicin uptake was determined by acquisition on a Guava EasyCyte 5 flow cytometer and results were analyzed using EasyCyte software (Millipore, Watford, UK).

### Statistical analysis

Statistics were performed using Minitab v18.1 (Minitab Ltd, Coventry, UK). Parametric statistics mean and standard error of the mean (SEM) were used for descriptive purposes and evaluation of significance was calculated by means of a two-tailed independent Student’s t-test. For graphical purposes in the figure captions: *p<0.05, **p<0.01 and ***p<0.001, whilst ‘ns’ denotes non-significance (p>0.05).

## Results

### The effect of anthracyclines doxorubicin and epirubicin on the viability of primary and immortalized human keratinocytes and the protective effects of cooling

**Fig 1:**
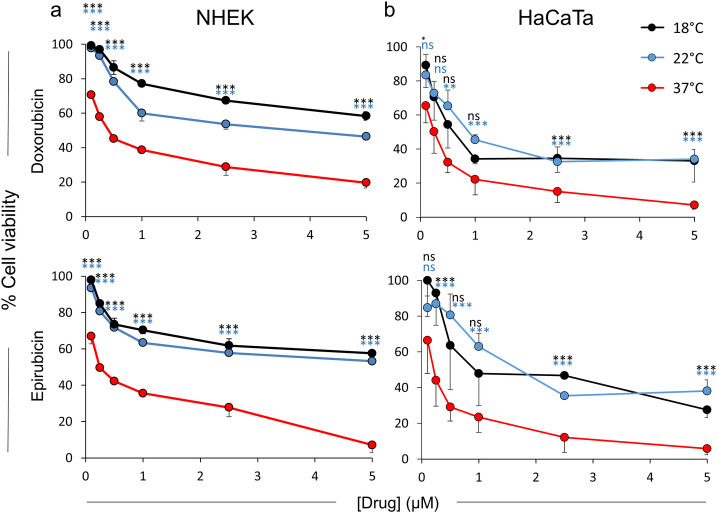
Cytoprotective effect of cooling against chemotherapy drug-mediated toxicity in NHEK and HaCaTa cells. NHEK (a) and HaCaTa (b) cells were treated for 2-hours with a range of concentrations ([drug]) of doxorubicin and epirubicin at normal (37°C) and cooling (22 and 18°C) conditions and cell viability was assessed 72-hours post-treatment. Data points correspond to mean % cell viability (± SEM) for independent biological experiments (n = 3), each consisting of 5 technical replicates. Statistical significance (cooling conditions *versus* 37°C) is denoted as *p<0.05, **p<0.01 and ***p<0.001, whilst ns denotes non-significance (p>0.05), with stats symbols being colour-coded for each cooling temperature.

We initially treated primary NHEK cultures and immortalized HaCaTa cells with doxorubicin and epirubicin at normal temperature conditions (37°C) and observed a dose-dependent reduction in keratinocyte viability (Fig 1). To mimic cooling conditions, we treated cells with the drugs at 22 or 18°C, which we have previously shown are cooling conditions that can protect such cells from chemotherapy drug-mediated cytotoxicity [13]. We found that cooling markedly protected from drug-mediated cytotoxicity for low drug doses (<1μM), whereas for high drug concentrations (>1μM) it significantly attenuated toxicity even at very high (5μM) doses (Fig 1). We confirmed these findings in similar experiments using the original HaCaT cells (S1 Fig). The lower temperature 18°C was consistently more effective at preventing toxicity particularly in primary NHEK cells, as was cooling-mediated cytoprotection overall in NHEK cultures in comparison to immortalized keratinocytes (Fig 1A and S1 Fig). These results confirmed our previous findings for doxorubicin [13], whilst for the first time extending our observations to the anthracycline epirubicin.

### Detection of anthracycline entry and localization in NHEK and HaCaTa cells and assessment of the effect of cooling in cellular drug uptake

**Fig 2:**
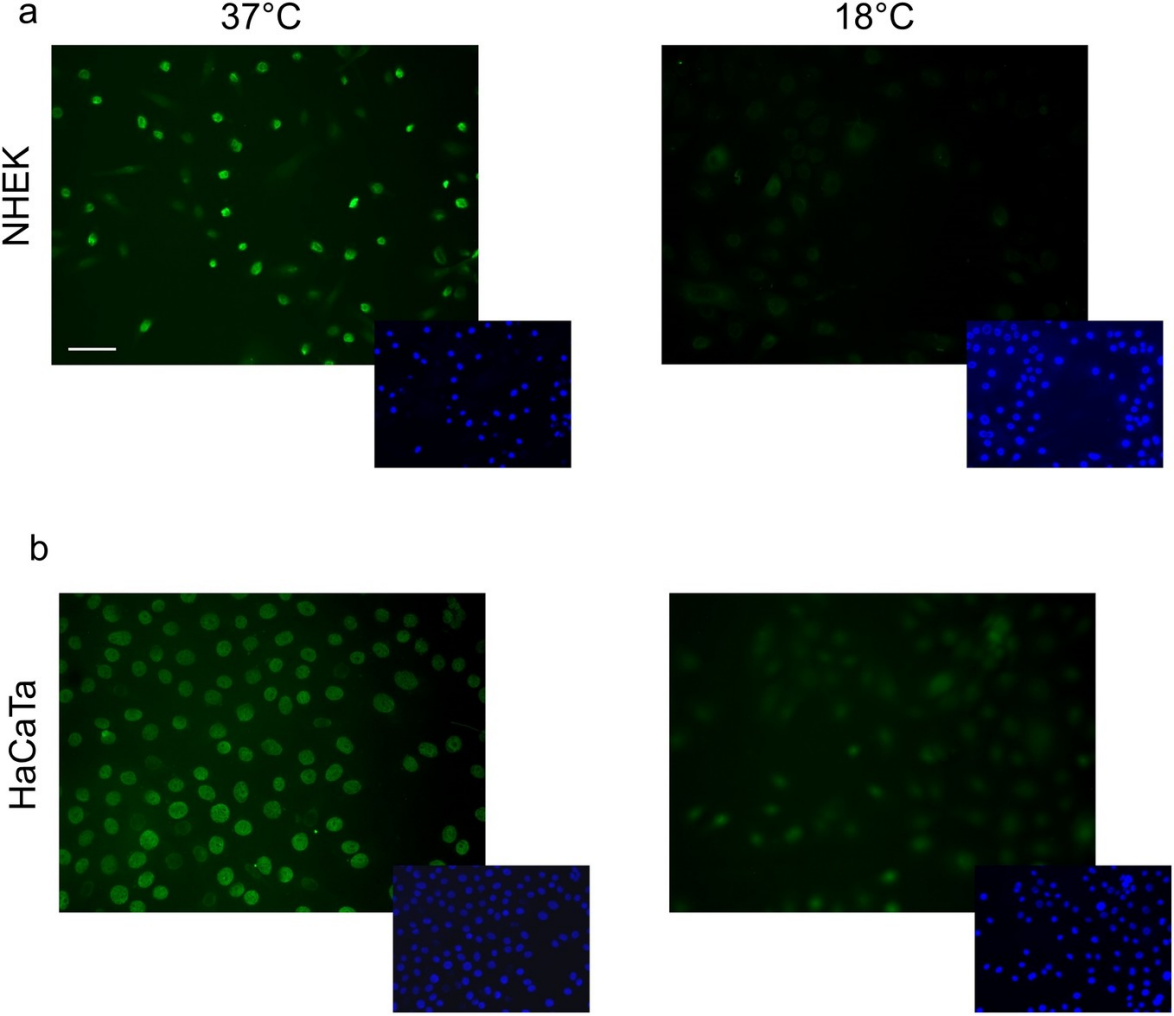
Determination of the effect of cooling on the cellular uptake of doxorubicin using fluorescence microscopy. NHEK (a) and HaCaTa (b) cells were treated with 1μM doxorubicin for 2-hours at normal (37°C) or cooling (18°C) conditions before being fixed and visualised by microscopy. Green fluorescence represents the presence of doxorubicin (larger images). Cell nuclei were visualised as blue by labelling with Hoechst 33258 following cell fixation (smaller images). Images are representative of results from three independent experiments. Scale bar: 50 μm.

To determine whether cooling rescues cells from chemotherapy drug-mediated cytotoxicity (at least partly) due to its ability to influence drug uptake, we exploited the inherent property of doxorubicin and epirubicin to fluoresce upon excitation and detected drug internalisation by microscopy. NHEK and HaCaTa cells were treated with doxorubicin at normal (37°C) or cooling (18°C) conditions and cellular drug uptake was determined by fluorescence microscopy (Fig 2). Doxorubicin was readily detected in treated NHEK and HaCaTa cells at 37°C and exhibited nuclear localization. Interestingly, when drug treatment was carried out under cooling conditions (18°C), there was little detectable doxorubicin in HaCaTa cells (Fig 2B) and even less in primary NHEK cultures (Fig 2A). When we performed similar experiments in NHEK and HaCaTa cells involving epirubicin, we observed epirubicin uptake and nuclear localization at normal temperature (37°C), which was suppressed under cooling (18°C) conditions (S2 Fig).

### Detection of anthracycline drug uptake inhibition by cooling in primary and immortalized human keratinocytes using flow cytometry

**Fig 3:**
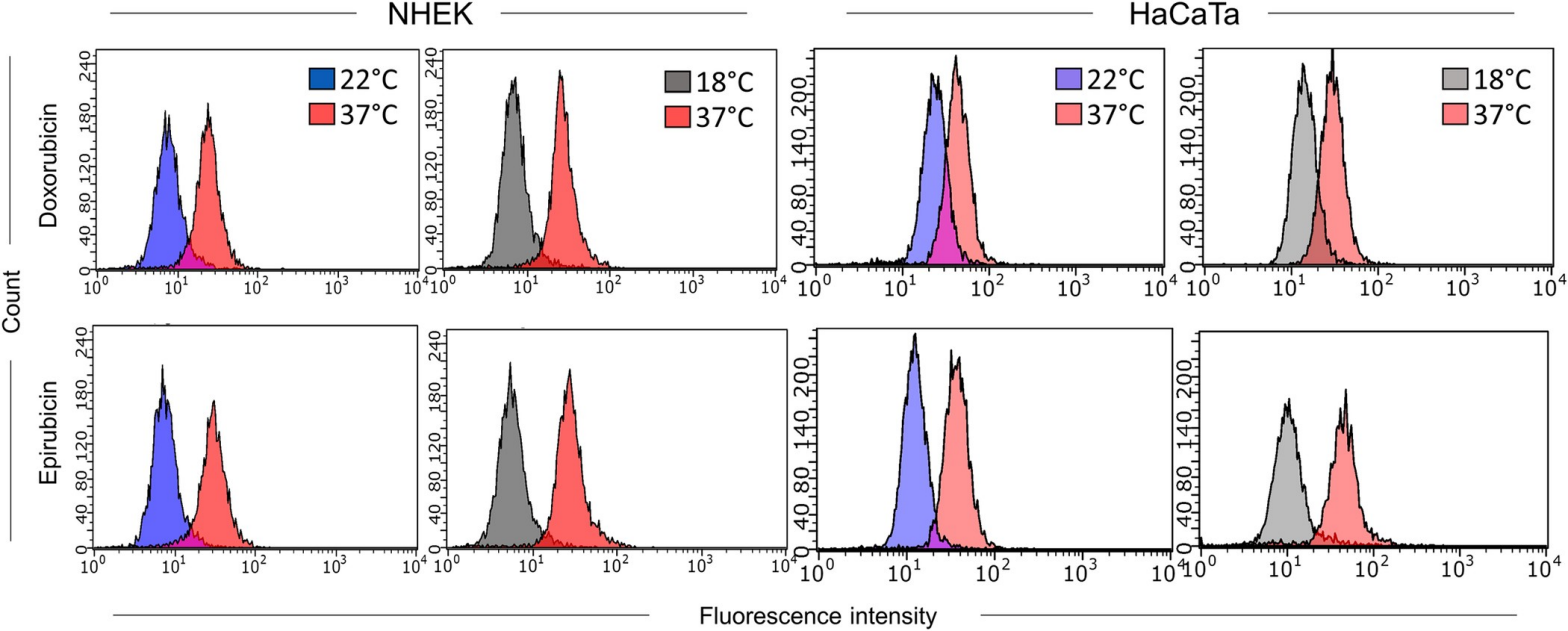
Effect of cooling on the cellular uptake of doxorubicin and epirubicin detected by flow cytometry. NHEK and HaCaTa cells were treated with 1μM doxorubicin (upper panels) or 1μM epirubicin (lower panels) for 2-hours at normal (37°C) and cooling (22 and 18°C) conditions before drug-associated fluorescence was assessed by flow cytometry. Overlay histograms represent log_10_ median fluorescence intensity for each temperature condition and in each cell type as indicated. Results shown are representative of three independent experiments.

To assess whether cooling attenuates cellular uptake of doxorubicin and epirubicin, we also exploited flow cytometry-based detection. A significant advantage of flow cytometry over microscopy is the ability to detect fluorescence more sensitively, as well as more readily quantify and compare cellular fluorescence for different conditions. Following treatment of primary NHEK cultures and HaCaTa cells with doxorubicin and epirubicin, drug uptake was readily detectable by flow cytometry at normal temperature (37°C); yet, cooling (22 and 18°C) caused clear reduction in cellular drug uptake (Fig 3). We confirmed these findings by performing similar experiments using the original HaCaT cell line (S3 Fig). Notably, the lower temperature 18°C was consistently more effective (than 22°C) at reducing the amount of detected fluorescence particularly in NHEK cells (as indicated by the greater ‘shift’ in the fluorescence histograms–Fig 3), which is in agreement with our observation that the temperature 18°C was more effective at preventing NHEK culture cytotoxicity (Fig 1).

### Determination of the extent of cooling-mediated reduction in cellular drug uptake by semi-quantitative flow cytometry-based analysis in primary and immortalized human keratinocytes

We then exploited the advantage of flow cytometry in measuring drug-associated fluorescence to devise a semi-quantitative approach that permitted determination of the differences in the amount of drug uptake at normal temperature (37°C) versus cooling conditions (22 and 18°C). The results obtained for this analysis of both doxorubicin and epirubicin were from three independent experiments performed using 2–3 internal replicates and carried out in both primary NHEK cultures as well as immortalized HaCaTa cells. In addition, all average median fluorescence intensity (MFI) values were calculated following ‘blank correction’, i.e. by subtraction of MFI values of non-treated (control) cells values from treated cultures to account for possible variability in cell auto-fluorescence due to differences in temperature.

**Fig 4:**
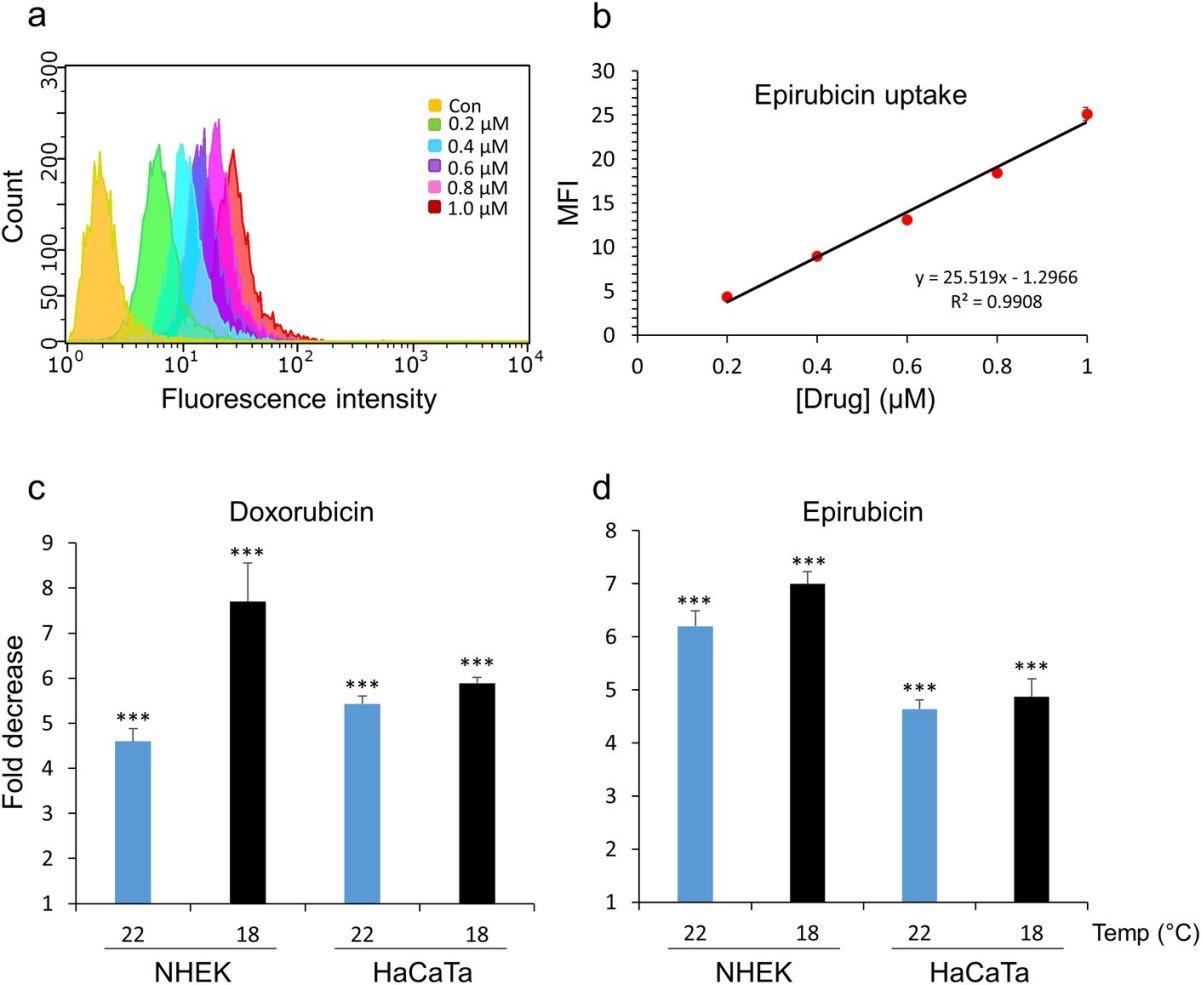
Flow cytometry-based quantification of the cellular uptake of doxorubicin and epirubicin at normal *versus* cooling conditions in normal and immortalized keratinocytes. a) NHEK cells were treated with the indicated doses of epirubicin (0.2–1.0μM) alongside untreated control (Con) cells at 37°C, before drug-associated fluorescence was detected by flow cytometry, as in Fig 3. The image is an overlay of the log_10_ median fluorescence intensity (MFI) histograms associated with each dose of epirubicin, as indicated. b) MFI values for independent replicate experiments (n = 3) performed as in (a) were plotted against the respective epirubicin concentration ([drug]) with the resulting equation from linear regression analysis included on the graph together with the R^2^ value. (c-d) The uptake of doxorubicin and epirubicin by NHEK and HaCaTa cells at normal (37°C) and cooling conditions (22 and 18°C) was determined by flow cytometry, before linear regression analysis was performed and fold decrease in drug uptake was calculated as explained in detail in the main text. Bars represent mean fold decrease (± SEM) in drug uptake for independent biological experiments (n = 3). Statistical significance (cooling condition *versus* 37°C) is denoted as ***p<0.001 for each temperature and cell type.

To quantify differences in intracellular drug concentration, we initially measured MFI values for cells treated with a series of defined doxorubicin and epirubicin concentrations at normal temperature (37°C). Representative fluorescence histograms from such experiments in NHEK cells are shown as overlay plots in S4 Fig (panels a and c) and the analysis of the epirubicin data set is presented in Fig 4, where MFI values for epirubicin concentrations 0.2, 0.4, 0.6, 0.8 and 1.0μM together with untreated controls (Fig 4A) were used to perform linear regression analysis. This identified a positive and linear correlation between [drug] (x-axis) and MFI (y-axis) (Fig 4B). A maximal drug dose of 1.0μM was used in order to maintain a linear relationship; above this concentration the detected fluorescence appeared to become saturated (not shown). For the data set shown in Fig 4A, the resulting equation is included on the graph (Fig 4B) together with the correlation coefficient (R^2^).

To determine differences in drug uptake mediated by cooling, we also performed a series of treatments of keratinocytes with a range of high doxorubicin and epirubicin concentrations, i.e. 1, 2, 3, 4 and 5μM carried out under cooling conditions (22 and 18°C) and measured cell fluorescence. Representative results from such experiments in NHEK cells at 18°C are shown in S4 Fig (panels b and d). We used the average MFI values obtained for each drug concentration under cooling conditions and based on each linear regression analysis (at 37°C) we calculated the equivalent drug concentration as follows:

$$\frac{Average\;MFI\;under\;cooling\;conditions}{Gradient\;of\;linear\;slope}=Calculated\;drug\;concentration\;equivalent\;to\;37{}^{\circ}C$$

This allowed us to then determine differences in cellular drug concentration by calculating the “fold decrease” in drug uptake as follows:

$$\frac{Actual\;drug\;concentration\;at\;cooling\;conditions}{Calculated\;drug\;concentration\;equivalent\;to\;37{}^{\circ}C}=Fold\;decrease\;in\;drug\;uptake$$

The data for all experiments in primary NHEK and HaCaTa keratinocytes are summarised in Fig 4C and 4D. It is noted that, rather than selecting specific doxorubicin and epirubicin concentrations to express representative fold decrease in drug uptake, individual fold differences were first calculated for each drug concentration, i.e. 1, 2, 3, 4 and 5μM, and a mean value for all of these concentrations was deduced and presented per cooling temperature studied (Fig 4C and 4D). We believe this provides a better quantification of the overall difference in drug uptake for the whole range of drug doses.

The data shows that cooling can reduce drug uptake by a minimum of ~4-fold, and this can increase up to ~8-fold (Fig 4C and 4D) depending on the cell model used or drug tested. We observed differences between cooling conditions, for instance cooling at 22°C reduced doxorubicin uptake in NHEK cultures by 4.6-fold whilst 18°C caused a further reduction to 7.7-fold (Fig 4C); similarly, cooling at 22°C reduced epirubicin uptake to 6.2-fold whilst 18°C caused a further reduction to 7-fold. Notably, therefore, the lower temperature 18°C caused more marked reduction in drug uptake (compared to 22°C) particularly in NHEK cells, once again in line with our observation that 18°C was consistently more effective at preventing NHEK culture cytotoxicity (Fig 1).

### Investigation of the influence of cooling on drug-mediated hair follicle-derived ORSK cytotoxicity and quantification of cooling-mediated reduction in drug uptake

**Fig 5:**
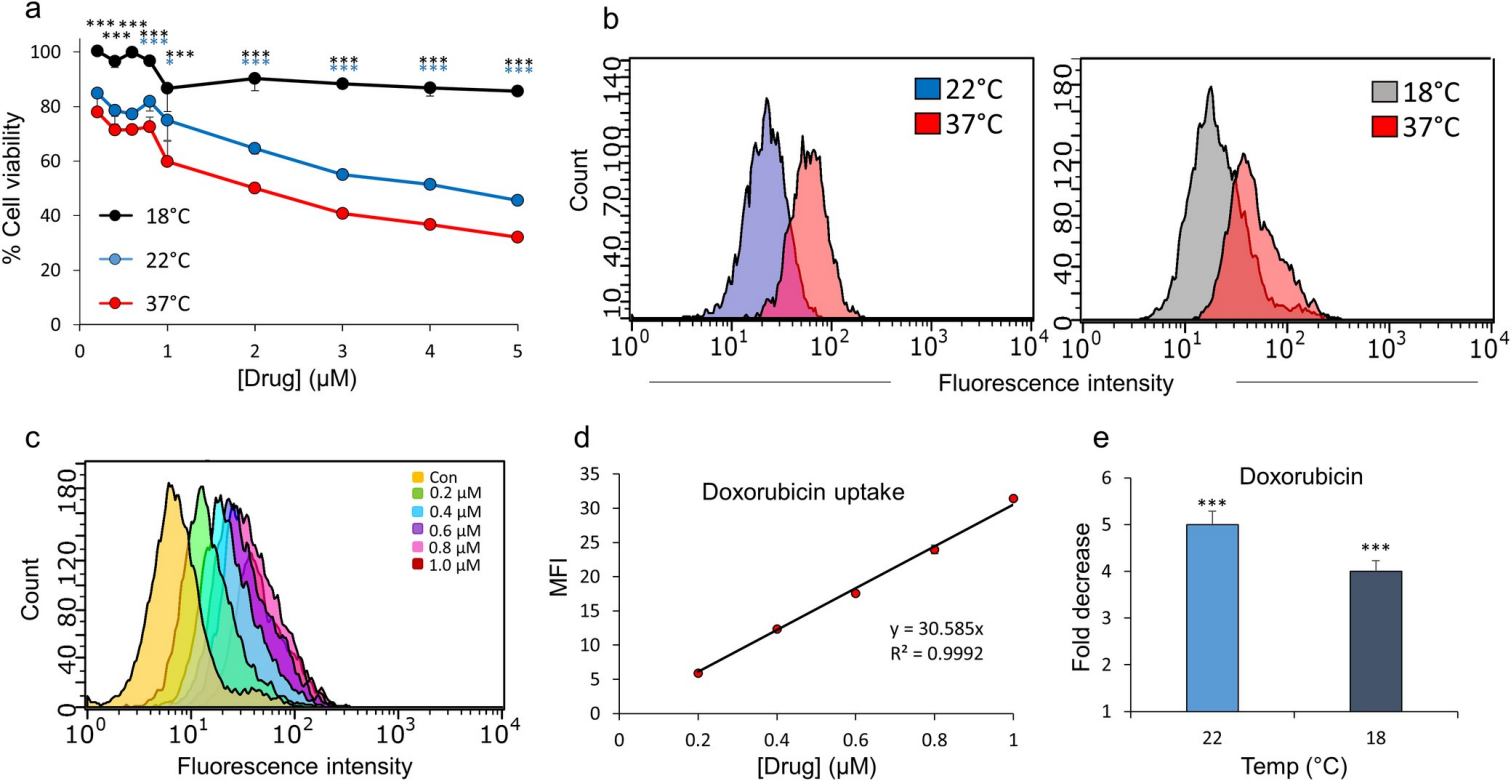
Cytoprotective effect of cooling against chemotherapy drug-mediated toxicity in hair follicle-derived keratinocytes and flow cytometry-based quantification of cellular doxorubicin uptake at normal versus cooling conditions. a) Hair follicle-derived ORSK cells were treated with a range of concentrations ([drug]) of doxorubicin at normal (37°C) and cooling (22 and 18°C) conditions and cell viability was assessed 72-hours post-treatment. Data points correspond to mean % cell viability (± SEM) for independent biological experiments (n = 3), each consisting of 5 technical replicates. Statistical significance (cooling conditions *versus* 37°C) is denoted as *p<0.05, **p<0.01 and ***p<0.001, with stats symbols being colour-coded for each cooling temperature. b) ORSK cells were treated with 1μM doxorubicin for 2-hours at normal (37°C) and cooling (22 and 18°C) conditions before drug-associated fluorescence was assessed by flow cytometry. Overlay histograms represent log_10_ median fluorescence intensity for each temperature condition as indicated. Results shown are representative of three independent experiments. c) ORSK cells were treated with the indicated concentration of doxorubicin (0.2–1.0μM) alongside untreated control (Con) cells at 37°C, before drug-associated fluorescence was detected by flow cytometry, as above (b). The image is an overlay of the log_10_ median fluorescence intensity (MFI) histograms associated with each dose of doxorubicin, as indicated. d) MFI values for independent replicate experiments (n = 3) performed as in (c) were plotted against the respective doxorubicin concentration ([drug]) with the resulting equation from linear regression analysis included on the graph together with the R^2^ value. e) The uptake of doxorubicin by ORSK cells at normal (37°C) and cooling conditions (22 and 18°C) was determined by flow cytometry, before linear regression analysis was performed and fold decrease in drug uptake was calculated as explained in detail in the main text. Bars represent mean fold decrease (± SEM) in drug uptake for independent biological experiments (n = 3). Statistical significance (cooling condition versus 37°C) is denoted as ***p<0.001 for each temperature.

Despite the robust and clinically-relevant observations obtained using primary and immortalized epidermal keratinocytes in this study and previously [13], these cells are not isolated from the keratinocyte niche of the HF. To circumvent this potential weakness in our approach, we employed previously reported methodologies [17, 19] to establish outer root sheath keratinocyte (ORSK) cultures as a more physiologically-relevant in vitro cell model. When we treated ORSK cultures with a range of concentrations of doxorubicin at 37°C, we observed dose-dependent reduction in cell viability (Fig 5A), however when ORSK cultures were treated with doxorubicin under cooling conditions (22 and 18°C), cooling markedly attenuated drug-mediated cytotoxicity. Specifically, cooling at 22°C caused a consistent reduction in cytotoxicity for low (<1μM) and high (>1μM) drug doses whilst, more strikingly, the 18°C temperature appeared to completely inhibit drug-induced cytotoxicity for the whole dose range, including high doses (5μM). These results are in concordance with our findings with NHEK cells, although clearly cooling at 18°C was effective in protecting ORSK cultures, and at high drug doses (Fig 5A) was more effective than was observed in NHEK (or even HaCaTa) cells (Fig 1A and 1B). Moreover, our findings extend our previous observations using commercially-available Human Hair Follicular Keratinocyte (HHFK) cultures treated with doxorubicin at 22°C [13], and reveal that an additional 4°C temperature reduction (18°C) renders cooling extremely effective at protecting against doxorubicin-mediated cytotoxicity in HF-derived keratinocytes.

ORSK cultures were then treated with doxorubicin to detect drug uptake by flow cytometry and assess the effect of cooling. Doxorubicin cellular uptake was readily detectable at normal temperature (37°C), and cooling (22 and 18°C) caused clear reduction in drug uptake (Fig 5B), although the lower 18°C temperature appeared to inhibit drug uptake less effectively than 22°C. We applied our semi-quantitative flow cytometry-based approach to carry out a linear regression analysis and determine the relative difference in drug uptake in ORSK cultures treated with doxorubicin at normal temperature (37°C) versus cooling conditions (22 and 18°C). By carrying out experiments with the same dose ranges as for the experiments with NHEK and HaCaTa cultures above (Fig 4), we first demonstrated a linear relationship between drug concentration and MFI values (Fig 5C and 5D). Then, by employing the experimental methodology and calculations detailed in the previous section, we utilised linear regression analysis to deduce fold differences in drug uptake in 37°C versus cooling conditions. We found that at 22°C ORSK cultures exhibited ~5-fold reduced doxorubicin uptake, whilst the reduction in drug uptake at 18°C was ~4-fold (Fig 5E). Our findings in ORSK cells confirmed that the ability of cooling to rescue dermal and follicular keratinocytes from anthracycline-mediated cytotoxicity directly coincided with its capacity to suppress cellular drug uptake. Yet, in ORSK cultures lowering the temperature from 22°C to 18°C did not cause any further reduction in drug uptake, despite the finding that 18°C was far more effective at protecting from drug-mediated cytotoxicity (Fig 5A).

## Discussion

Scalp cooling represents the only available treatment against CIA [7], but its mechanisms of cytoprotection remain unknown. In this study we have systematically explored for the first time the hypothesis that cooling reduces the entry of chemotherapy drugs in human keratinocytes. We used primary NHEK cultures as well as immortalized HaCaTa, alongside HF-derived ORSK cultures and assessed different temperatures, by studying doxorubicin and epirubicin. Doxorubicin can be detected by fluorescence microscopy in mammalian cells and that it diffuses primarily to the nucleus [15], and flow cytometry-based detection has been used [24]. Detection of epirubicin by flow cytometry and nuclear localization by microscopy have also been reported [16, 25]. The main mechanism of action of doxorubicin is DNA intercalation and genotoxic damage consistent with its nuclear localization [26].

We have demonstrated that doxorubicin and epirubicin are concentrated in the nuclei of NHEK and HaCaTa cells and are highly cytotoxic at 37°C; however, cooling markedly reduced chemotherapy drug entry, which coincided with its ability to reduce or prevent drug-mediated keratinocyte cytotoxicity. We showed reduction in drug uptake and nuclear localization qualitatively by fluorescence microscopy, and also devised a methodology for semi-quantitative analysis of differences in drug uptake at different culture (temperature) conditions using flow cytometry. The approach allowed us to determine that cooling can reduce drug uptake by up to ~8-fold, with cooling to 18°C showing a stronger ability (compared to 22°C) to reduce drug uptake in NHEK and HaCaTa cells, in line with our observations on the effects of cooling on drug-mediated cytotoxicity.

The way by which cooling reduces drug uptake remains unknown. In fact, it is still to be established by what mechanism anthracyclines such as doxorubicin and epirubicin move across the plasma membrane. As both drugs are moderately lipophilic (logP of doxorubicin is 1.27 and epirubicin 1.41), they could at least to some extent penetrate the phospholipid bilayer, as confirmed for doxorubicin in model phospholipid membranes [27]. Intracellular accumulation of doxorubicin was reported in pig kidney LLC-PK1 cells even after inhibition of drug transporters [28]. However, as with other anthracyclines, doxorubicin cellular uptake can be mediated by transport proteins, the organic anion-transporting proteins SLC22A16 and OATP1A2, OATP1B1, OATP1B3 [29], thus expression of these in the HF cell niche could enhance the uptake of doxorubicin into the cell by an active process. Nevertheless, irrespective of how anthracyclines enter the cell, uptake is expected to be temperature-modulated as passive permeability of compounds across phospholipid bilayers is affected by temperature. Indeed, a 2-fold increase in doxorubicin permeability was observed when culture temperature was increased from 37°C to 47°C in CHO cells [30] and in Sarcoma-180 cells [31]. By contrast, reducing the temperature in LLC-PK1 cells from 37°C to 4°C resulted in a 4.5-fold reduction in doxorubicin uptake in the absence of any evidence for active drug uptake [28]. If either doxorubicin or epirubicin are substrates for one or more specific transport protein(s) that facilitate(s) cellular entry, one could assume that lowering the metabolic rate of cells by cooling would lead to a reduction in the transporter mediated drug uptake. Yet, previous studies have shown that doxorubicin entry shows no signs of plateau [32] and passive diffusion appeared to be the main mode of transport [33]. Equally importantly, the latter study provided evidence for temperature-dependent structural changes to the plasma membrane, with changes being observed at temperatures below 22°C [33].

Interestingly, and perhaps unexpectedly, in ORSK cultures lowering the temperature from 22°C to 18°C did not cause any further reduction in drug uptake, despite that 18°C was far more effective at protecting from doxorubicin cytotoxicity. We believe that these observations are not in disagreement with the results from NHEK cultures. Instead, the findings using this more physiological-relevant cell type provide evidence that, although cooling is protective and this property coincides with reduction in drug uptake, blockade of drug uptake is most probably not the only cooling-associated mechanism and it operates in concert with other mechanisms; for instance, mild hypothermia has been reported to be cytoprotective against a variety of cytotoxic stimuli [34]. We believe that the ability of cooling to cytoprotect is underpinned by a combination of mechanisms, which may include reduction in cell division rate and attenuation of enzymatic pathways that mediate cytotoxicity (apoptosis/necrosis).

Our flow cytometry-based semi-quantitative detection system has been a useful tool in determining differences in anthracycline uptake in human keratinocytes and shows a high level of sensitivity. Our analysis could distinguish between intracellular doxorubicin and epirubicin concentrations as little as 0.1μM and with consistency and sensitivity across a range of keratinocyte types (primary and immortalized). We believe this system could also be exploited in other contexts to determine anthracycline drug entry in other cell types whether normal or malignant. For instance, anthracycline-mediated cardiovascular toxicity [35] and cardiomyopathy [36] represent an important area of research, with doxorubicin in particular causing long-term toxicity and cardiomyopathy [37]. Thus, our flow cytometry-based approach may permit measurements of drug uptake in cell types, such as primary human cardiomyocytes or vascular endothelial cells, as well as offering the opportunity to test strategies that can reduce drug uptake/toxicity and confirm their potential efficacy.

The ability of cooling to attenuate cellular drug uptake represents at least one of the mechanisms that underpin the clinical efficacy of scalp cooling in reducing or even preventing CIA by protecting cells from the toxicity of chemotherapy drugs. It is tempting to speculate that the ability of cooling to reduce drug uptake is not limited to anthracyclines as shown here, but also extends to other types of chemotherapy drugs, although further studies would be necessary to address this hypothesis.

## Supporting information

S1 Fig Cytoprotective effect of cooling against chemotherapy drug-mediated toxicity in HaCaT cells. HaCaT cells were treated with a range of concentrations ([drug]) of doxorubicin and epirubicin at normal (37°C) and cooling (22 and 18°C) conditions and cell viability was assessed 72-hours post-treatment. Data points correspond to mean % cell viability (± SEM) for independent biological experiments (n = 3), each consisting of 5 technical replicates.
(TIF)

S2 Fig Detection of the effect of cooling on the cellular uptake of epirubicin using fluorescence microscopy. NHEK (a) and HaCaTa (b) cells were treated with the indicated concentration of epirubicin for 2-hours at normal (37°C) or cooling (18°C) conditions before being visualised by live fluorescence microscopy. Green fluorescence represents the presence of epirubicin. Images of untreated NHEK and HaCaTa cells (denoted ‘Control’) were included. Scale bar: 50 μm.
(TIF)

S3 Fig Effect of cooling on the uptake of doxorubicin and epirubicin in HaCaT cells detected by flow cytometry. HaCaT cells were treated with 1μM doxorubicin or 1μM epirubicin for 2-hours at normal (37°C) and cooling (22 and 18°C) conditions before drug-associated fluorescence was assessed by flow cytometry. Overlay histograms represent log_10_ median fluorescence intensity for each temperature condition as indicated. Results shown are representative of three independent experiments.
(TIF)

S4 Fig Flow cytometry-based detection of the cellular uptake of low and high doses of epirubicin and doxorubicin at normal *versus* cooling temperature conditions in normal keratinocytes. NHEK cells were treated with the indicated concentrations of i) epirubicin alongside untreated control (0.0μM) cells at 37°C (a) and 18°C (b), or ii) doxorubicin alongside controls (0.0μM) at 37°C (c) and 18°C (d). Drug-associated fluorescence was detected by flow cytometry. The panels represent overlays of the log_10_ median fluorescence intensity (MFI) histograms obtained for each drug dose, as indicated. Results are representative of three independent experiments.
(TIF)

## Abbreviations

CIA: chemotherapy-induced alopecia
FBS: fetal bovine serum
HaCaTa: adapted HaCaT
HDF: human dermal fibroblasts
HF: hair follicle
HKGS: Human Keratinocyte Growth Supplement
KSFM: keratinocyte serum-free medium
MFI: median fluorescence intensity
NHEK: normal human epidermal keratinocytes
ORSK: outer root sheath keratinocytes
