# Supplementary figures and images for: Cooling-mediated protection from chemotherapy drug-induced cytotoxicity in human keratinocytes by inhibition of cellular drug uptake
Source: PLoS One. 2020 Oct 15;15(10):e0240454. doi: 10.1371/journal.pone.0240454 (PMC7561111; doi:10.1371/journal.pone.0240454)

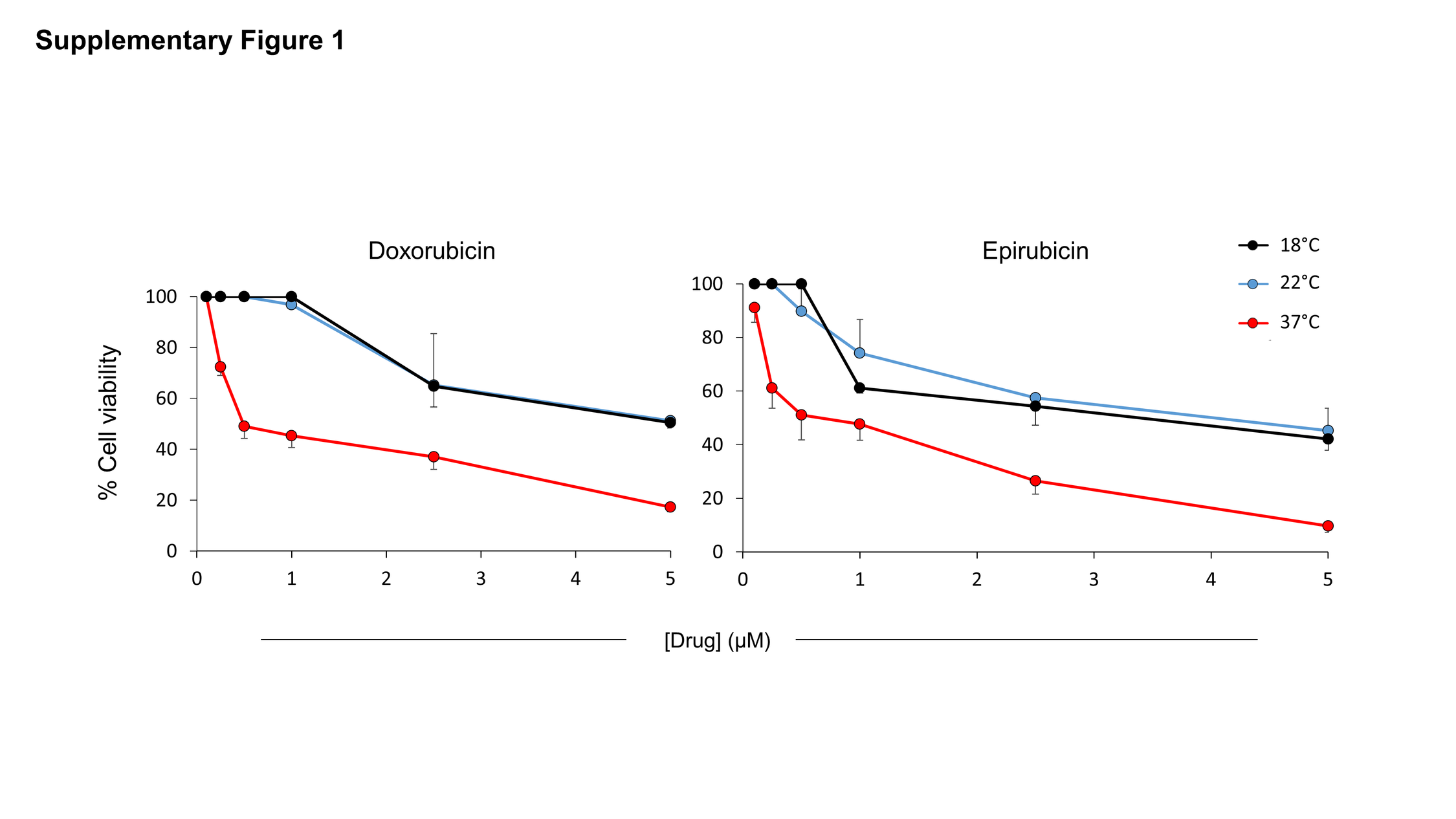

Supplement: S1 Fig — HaCaT cells were treated with a range of concentrations ([drug]) of doxorubicin and epirubicin at normal (37°C) and cooling (22 and 18°C) conditions and cell viability was assessed 72-hours post-treatment. Data points correspond to mean % cell viability (± SEM) for independent biological experiments (n = 3), each consisting of 5 technical replicates. (TIF) [file pone.0240454.s001.tif]

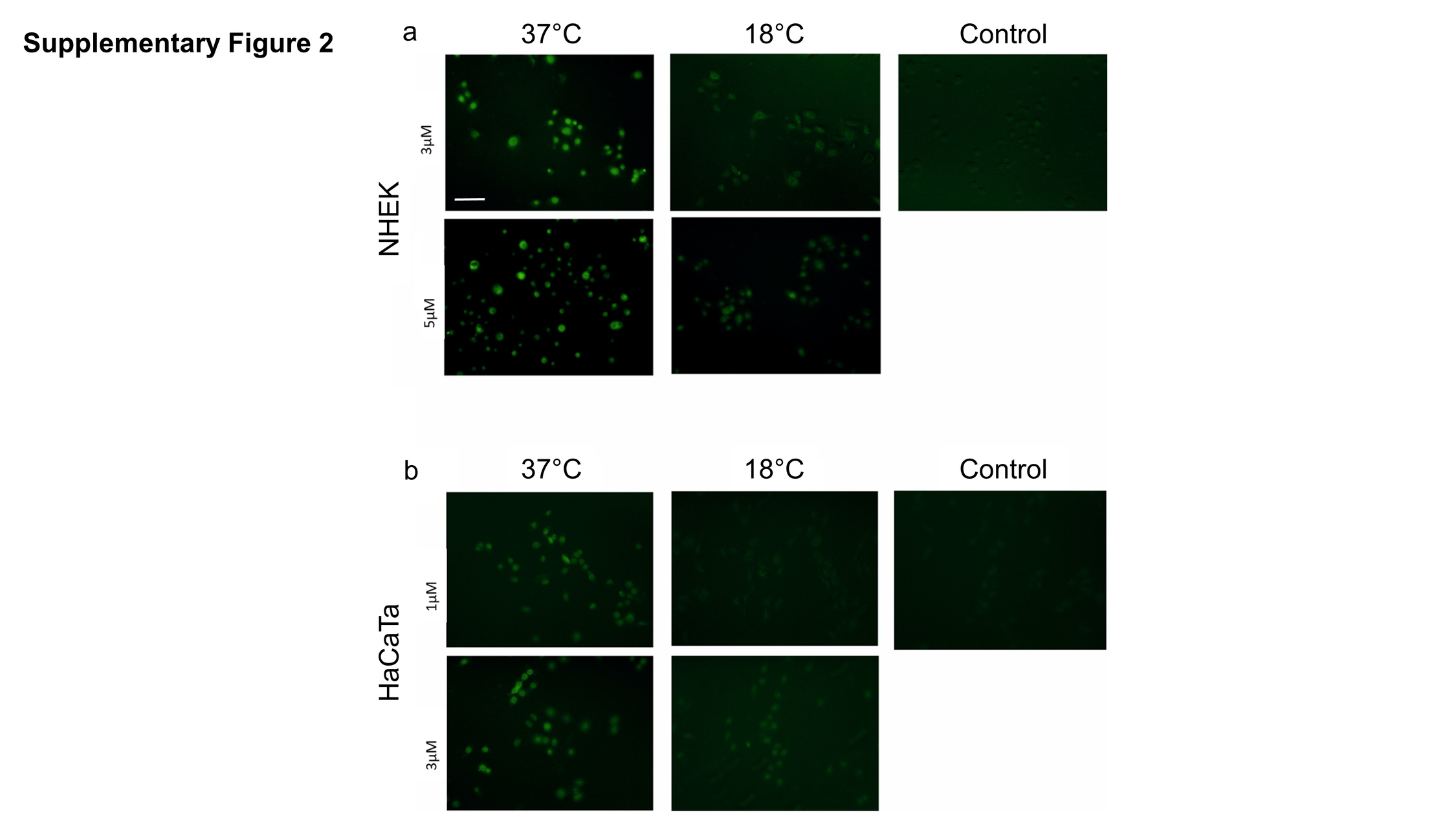

Supplement: S2 Fig — NHEK (a) and HaCaTa (b) cells were treated with the indicated concentration of epirubicin for 2-hours at normal (37°C) or cooling (18°C) conditions before being visualised by live fluorescence microscopy. Green fluorescence represents the presence of epirubicin. Images of untreated NHEK and HaCaTa cells (denoted ‘Control’) were included. Scale bar: 50 μm. (TIF) [file pone.0240454.s002.tif]

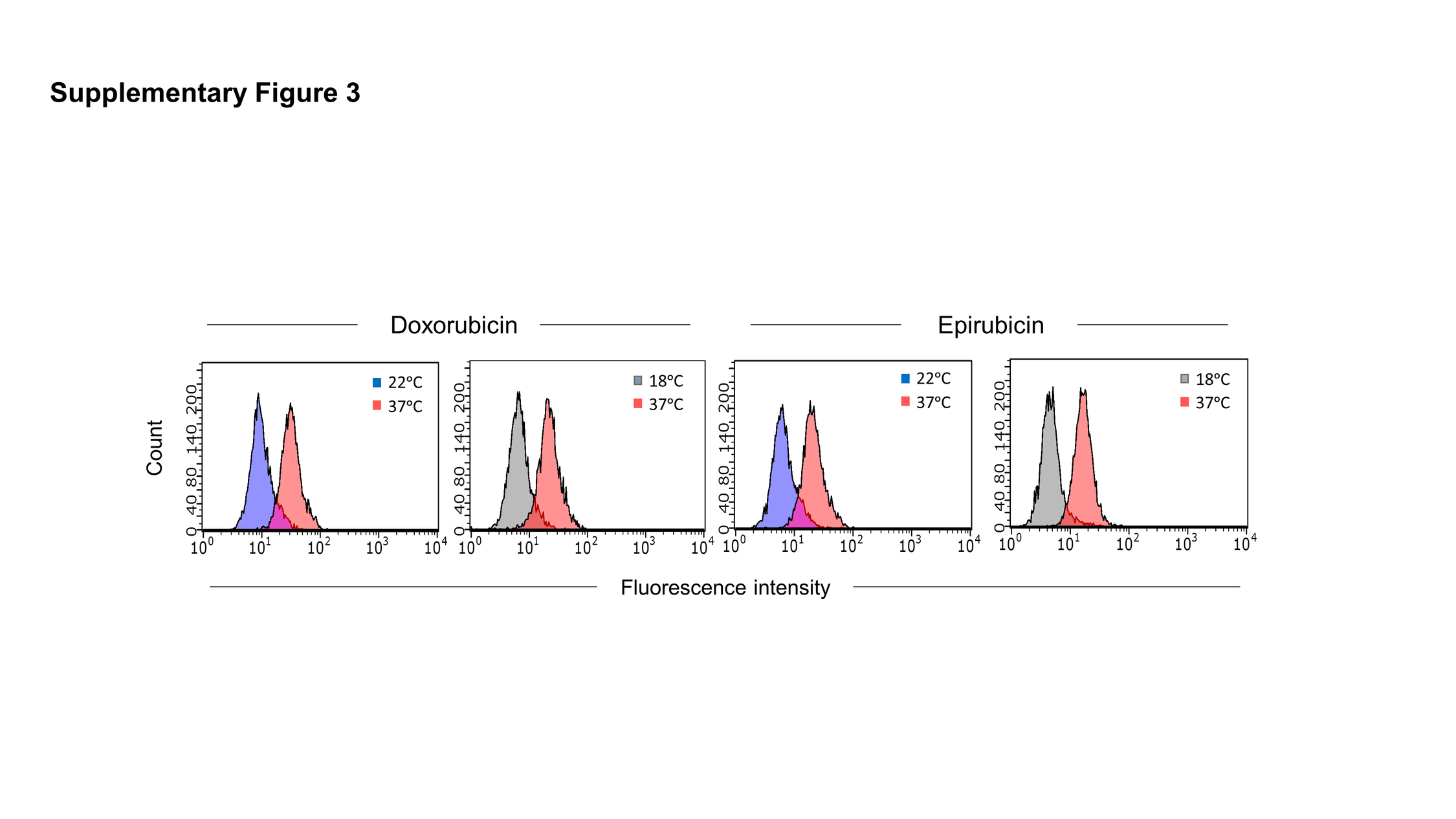

Supplement: S3 Fig — HaCaT cells were treated with 1μM doxorubicin or 1μM epirubicin for 2-hours at normal (37°C) and cooling (22 and 18°C) conditions before drug-associated fluorescence was assessed by flow cytometry. Overlay histograms represent log10 median fluorescence intensity for each temperature condition as indicated. Results shown are representative of three independent experiments. (TIF) [file pone.0240454.s003.tif]

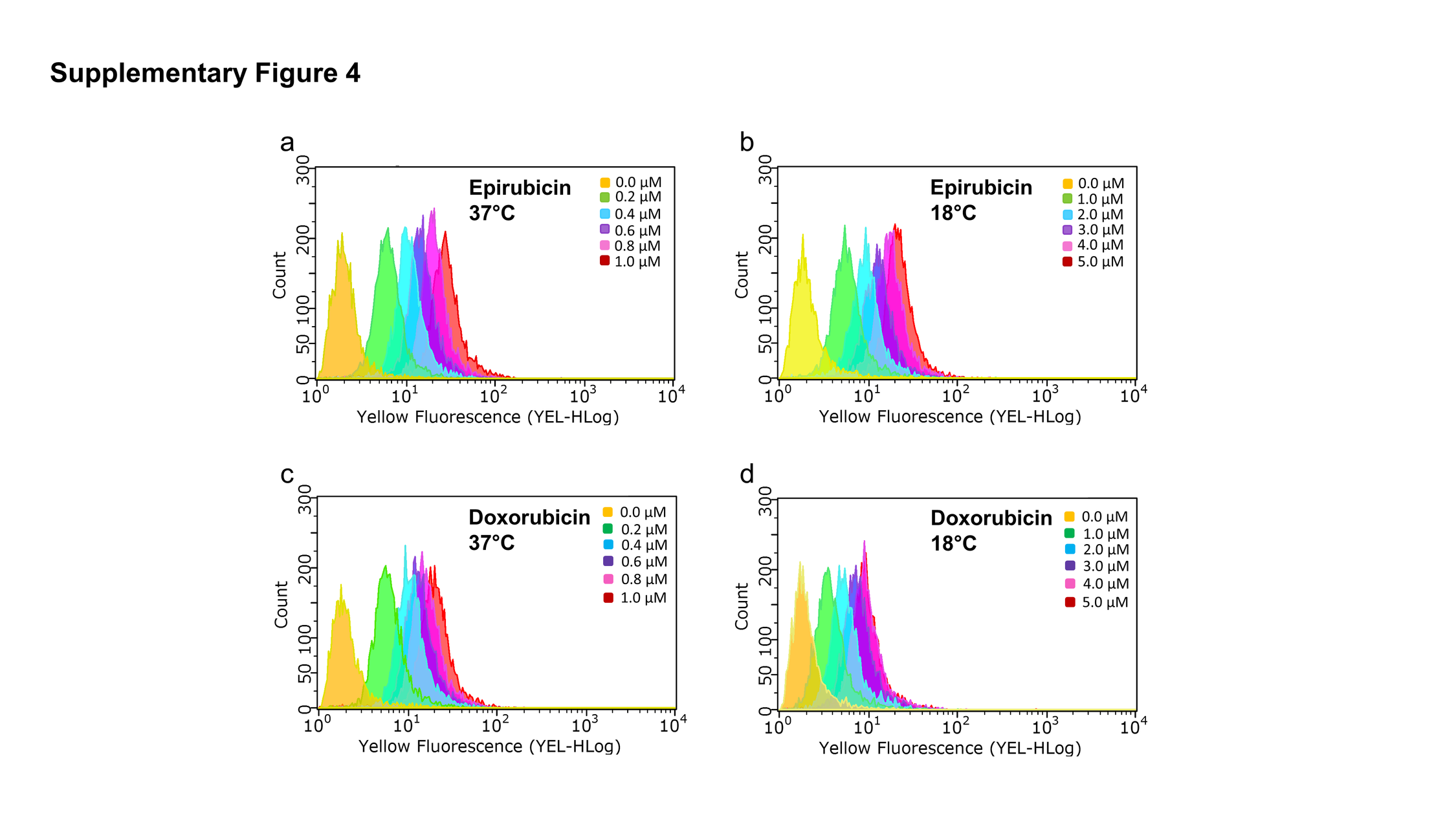

Supplement: S4 Fig — NHEK cells were treated with the indicated concentrations of i) epirubicin alongside untreated control (0.0μM) cells at 37°C (a) and 18°C (b), or ii) doxorubicin alongside controls (0.0μM) at 37°C (c) and 18°C (d). Drug-associated fluorescence was detected by flow cytometry. The panels represent overlays of the log10 median fluorescence intensity (MFI) histograms obtained for each drug dose, as indicated. Results are representative of three independent experiments. (TIF) [file pone.0240454.s004.tif]
